# Revealing the Complex Relationship Among Hyperspectral Reflectance, Photosynthetic Pigments, and Growth in Norway Spruce Ecotypes

**DOI:** 10.3389/fpls.2022.721064

**Published:** 2022-05-30

**Authors:** Jakub Hejtmánek, Jan Stejskal, Jaroslav Čepl, Zuzana Lhotáková, Jiří Korecký, Anna Krejzková, Jakub Dvořák, Salvador A. Gezan

**Affiliations:** ^1^Department of Genetics and Physiology of Forest Trees, Faculty of Forestry and Wood Sciences, Czech University of Life Sciences Prague, Prague, Czechia; ^2^Department of Experimental Plant Biology, Faculty of Science, Charles University in Prague, Prague, Czechia; ^3^VSN International, Hemel Hempstead, United Kingdom

**Keywords:** Norway spruce [*Picea abies* (L.) H. Karst], hyperspectral reflectance, chlorophyll, ecotypes genetic variation, genetic correlation, broad-sense heritability

## Abstract

Norway spruce has a wide natural distribution range, harboring substantial physiological and genetic variation. There are three altitudinal ecotypes described in this species. Each ecotype has been shaped by natural selection and retains morphological and physiological characteristics. Foliar spectral reflectance is readily used in evaluating the physiological status of crops and forest ecosystems. However, underlying genetics of foliar spectral reflectance and pigment content in forest trees has rarely been investigated. We assessed the reflectance in a clonal bank comprising three ecotypes in two dates covering different vegetation season conditions. Significant seasonal differences in spectral reflectance among Norway spruce ecotypes were manifested in a wide-ranging reflectance spectrum. We estimated significant heritable variation and uncovered phenotypic and genetic correlations among growth and physiological traits through bivariate linear models utilizing spatial corrections. We confirmed the relative importance of the red edge within the context of the study site’s ecotypic variation. When interpreting these findings, growth traits such as height, diameter, crown length, and crown height allowed us to estimate variable correlations across the reflectance spectrum, peaking in most cases in wavelengths connected to water content in plant tissues. Finally, significant differences among ecotypes in reflectance and other correlated traits were detected.

## Introduction

Norway spruce (*Picea abies* L. Karst) is one of Europe’s most dominant tree species of forest ecosystems. It is the most common coniferous tree species of European forests and has a wide nature range from the French Alps (5°E) to the Ural Mountains (155°E; Oleksyn et al., 1998). The altitudinal range is also very wide: Norway spruce original occurrence stretches from the sea level 0–2,300 m in the Italian Alps (Jansson et al., 2013). In recent years, the climate has changed rapidly, impacting the microclimate in forests and the water regime in general. The average annual temperature also has risen slightly (Karlsson et al., 1997; Wallin et al., 2002). It is well known that Norway spruce is relatively sensitive, especially to nutrient deficiencies and soil water content. Thus, it is not surprising that recent years’ observations show drought stress impacts on *P. abies* (Ditmarová et al., 2010). Some consequences of climate change, such as increased temperature and acute drought events, cause a global problem with bark beetles in forests. Bark beetles are the most devastating biotic agents in many forest ecosystems (Anderegg et al., 2015). The higher frequency of dry summers and warmer temperatures is an important predisposing factor triggering beetles’ outbreaks and affecting population dynamics (Jactel et al., 2012; Weed et al., 2013; Hart et al., 2014).

*Picea abies* is a polymorphic tree species, and its taxonomic variation is a highly discussed topic (Tutin et al., 1964). There are two main taxonomic types: *P. abies* var.*abies*—European spruce and *Picea abies* var.*obovata*—Siberian spruce. These two types have significant genetic similarities, suggesting that they belong to the same species (Krutovskii and Bergmann, 1995). Morgenstern (1996) described three morphological forms (ecotypes) of Norway spruce. These forms are low-elevation (naturally growing in areas up to 500 m), medium-elevation, and high-elevation (above 1,100 m a.s.l.). The most significant difference lies in the crown morphology. Low-elevation (*acuminata*) has a wide crown with long branches, while high-elevation (*obovata*) has a narrow crown with short branches pointing down. Genetic differences among the putative ecotypes were recently confirmed by Korecký et al. (2021). There are emerging pieces of evidence that some physiological traits also differ among the ecotypes, Tomášková et al. (2021) found that parameters describing structural indicators of PSII in the thylakoid membrane (calculated from the OJIP test) distinguished *obovata* ecotype. Androsiuk et al. (2013) and Farjon and Filer (2013) also reported specific adaptations for the given geographic area, climatic area, and forest vegetation zone for each ecotypic form. Almost every investigated trait is closely connected to local conditions. Norway spruce is relatively sensitive to drought stress, and this stress is related to adaptations to climate changes (Van der Maaten-Theunissen et al., 2013). High variation in drought stress within Norway spruce populations across vegetation zones was previously reported (Trujillo-Moya et al., 2018). Recently, a significant variation among Norway spruce ecotypes was found in dehydrin gene expression (Čepl et al., 2020). The most notable difference between acuminata and obovata ecotype was found in specific dehydrin PaCAP1 expression. PaDhn4,5 and PaDhn6 dehydrins genes strongly correlate with a climatic variable such as temperature and precipitation.

In general, water deficit is an essential source of abiotic stress with complex effects on plants, including many physiological and biochemical responses, leading to inhibited growth or even mortality (Farooq et al., 2009; Harb et al., 2010). The seedlings experiments with Norway spruce showed a significant decrease in total chlorophyll concentration during advanced stages of dehydration (Ditmarová et al., 2010). These results demonstrate that the drought response of *P. abies* causes many biochemical changes, and the shifts in selected compounds’ content can be used as an indicator for water stress (Ditmarová et al., 2010). Degradation and content of chlorophylls and proline levels could be used as biomarkers for an early assessment of water stress of Norway spruce. Specific populations are more sensitive to water stress, which indicates some tolerance to osmotic stress during seed germination (Schiop et al., 2017). All photosynthetic pigments, mostly carotenoids, play some role in drought tolerance (including oxidative damage caused by water deficit). Thus, increased carotenoid content is an important indicator of water stress tolerance (Jaleel et al., 2009).

Hyperspectral reflectance of foliage or canopy reveals information connected to its biochemical composition, water content, and structure and thus to plants’ health status (Campbell et al., 2004; Kokaly et al., 2009; Ustin et al., 2009). This valuable information, often hidden from human eyes, has been utilized in many research fields in past decades (Goetz, 2009; Masaitis et al., 2013). Reflectance features in the visible (VIS) part of the electromagnetic spectrum are primarily determined by photosynthetic pigments’ content (Gates et al., 1965). The VIS spectral region is most usually used to evaluate leaf and canopy phenology (Junker and Ensminger, 2016; Yang et al., 2016) and physiological status (Carter, 1993; Mišurec et al., 2012; Einzmann et al., 2021). The sharp increase in reflectance around 700 nm due to the strong chlorophyll absorption band centered around 680 nm coupled with a scattering of near-infrared wavelengths within the leaf is known as “the red edge” (Curran et al., 1990; Gitelson et al., 1996). The red edge (RE) spectral region is also often used for vegetation stress detection. The inflection point of the reflectance curve within the red edge may shift to lower wavelengths due to a decrease of chlorophyll content in leaves or needles (Rock et al., 1988; Curran et al., 1990). The environmental stress factors causing this so-called blue shift of inflection point could be various: worsening of the tree physiological status due to air pollution (Campbell et al., 2004), decreased tree vitality after artificial ring-barking (girdling), and bark beetle infestation (Einzmann et al., 2021) and many others (viral infection, trace metal accumulation; Rathod et al., 2018; Golhani et al., 2019). One crucial question is whether the reflectance in this biologically important spectral region exhibits a similar shift across the studied ecotypes and if this shift can also be observed across the vegetation season. The position of the red edge inflection point is sensitive to environmental factors, but it may also be genetically conditioned. Heritable variation in the red edge was described within the Scots pine population by Čepl et al. (2018). The reflectance in the near-infrared spectral region provides information about leaf structure at the leaf level (Gates et al., 1965; Slaton et al., 2001) and the water content at leaf and canopy level (Kokaly et al., 2009). Vegetation indices (VIs) are various spectral transformations that reduce raw multivariate spectral data to single index values (Jiang et al., 2018; Verrelst et al., 2019). Plenty of VIs was defined^1^ for leaf and canopy reflectance level. According to the reflectance wavelengths they are computed from, the VIs may be used for retrieval of several leaf traits—usually chlorophylls (Croft et al., 2014; Neuwirthová et al., 2021), anthocyanins (Gitelson et al., 2009), nitrogen (Berger et al., 2020), lignin (Serrano et al., 2002), and water content (Hunt and Rock, 1989; Peñuelas et al., 1993). Regarding population variability in hyperspectral reflectance (Cavender-Bares et al., 2016), showed that multivariate spectral information could distinguish populations of the same species more accurately than individual leaf functional traits.

Our study investigates the differences in the shoot hyperspectral reflectance among three Norway spruce ecotypes and how this variation manifests in a wide-ranging reflectance spectrum. We hypothesized that shoot spectral signal would reflect ecotypes’ adaptation to the local environment of origin and thus allow us to distinguish the ecotypes even after acclimation to conditions of the common-garden experiment. We used the well-established vegetation indices not as simple predictors of given biophysical traits but as a trait that can be genetically determined. Traditionally, the spectral vegetation indices are constructed or pre-selected due to specific absorption features of biochemical compounds or structural traits connected to a stress reaction or health status. With this in mind, we chose heritable vegetation indices from the original candidate set contained within the R package hsdar (Lehnert et al., 2019). The heritable indices subsequently entered the bivariate analysis, which allowed us to estimate genetic correlations with other traits of interest. We estimated phenotypic and genetic correlations among growth and physiological traits to verify their complex relationship through bivariate models and spatial analysis. Subsequently, we examined phenotypic and genetic correlations of the established reflectance indices with growth traits and photosynthetic pigment content. In connection with the aims mentioned above, we confirmed the relative importance of the red edge within the context of the study site’s ecotypic variation. We observed that the phenotypic/genetic correlations vary across the reflectance spectrum, peaking mostly in wavelengths related to chlorophyll and water content in plant tissues.

## Materials and Methods

### Plant Material

This experiment comprises a unique clonal Norway spruce common-garden experiment established in the Czechia (N 49°56.37′, E 14°20.96′) in 1970. Vegetatively propagated material used for this experiment originates from several Czech Norway spruce populations. Grafted trees were planted as clonal rows. This trial is unique because it represents all the morphotypes of Norway spruce, which occur in this country. These morphological forms (putative ecotypes) retain some characteristic features, which correspond to their altitudinal origin. Genotypes of the low-elevation form (*acuminata*) originated from the altitude 360 m a.s.l., medium-elevation altitude form (*europaea*) from the altitude 770–775 m a.s.l, and the high-elevation form (*obovata*) from the altitude 1,145–1,175 m a.s.l. The exact geographical origin of the sampled ecotypes (grafting locations) is shown in Supplementary Figure 1.

### Trial Description

This experimental trial is situated in low relief of 320–340 m a.s.l. It was established in 1970 (Šindelář, 1975). Bedrock constitutes clayey Algonkian phyllite slates with variously thick loess and sloping clay overlaps. The soils can be characterized as medium-deep cambisols in strongly skeletal bases, in places with signs of reduction processes. The upper horizons are clayey, the lower horizons heavier, silty clay. There is a lack of loess cover in areas, and the soils are generally richer in the skeleton (particles >2 mm). The average tree height on the plot (2020) was 20.6 m; sd = 2.95 m, and the average DBH was 33.1 cm; sd = 7.7 cm.

### Sampling

One branch (30 cm) per tree was sampled with telescopic pruning poles. We targeted needles from the crown’s southern exposure, 5–8 m above ground, corresponding to the transition or shaded crown part. Shoot samples were collected from 86 (87 in August) trees in two periods. The first one was realized on May 25th, 2020, and the second sampling was on August 18th, 2020. In May, the current year’s needles were not yet fully developed; therefore, the previous-year needles were collected for reflectance analysis. In August, the current year needles were already mature. However, in some trees, the terminal buds remained dormant, and thus the mixed sample of current and previous-year needles was taken for reflectance measurement and pigment analyses. The sampling procedure consisted of a random selection of replicated clones from three represented ecotypes, ensuring that we covered different microsites within the trial. All sampled trees retained their morphological characteristics (ecotypic appearance) correspondingly to their altitudinal origin. These two periods were chosen concerning actual climatic conditions, especially the measure of water stress of trees. The experimental layout and the highlighted sampled trees are shown in Supplementary Figure 1. In addition, we measured standard growth parameters such as tree height (m) and diameter in breast height (DBH; cm). Subsequently, we measured the bottom height of the vital crown (HC) and the overall length of the crown (LC) for each tree.

### Climatic Data

Climatic data for this study were obtained from the nearest meteorological station (Praha- Libuš). Ten kilometers from the test site. We reported the climatic data 14 days before sampling. Before the first sampling, the mean daily temperature was 12.4°C, and before the second sampling 22.6°C. Mean daily precipitation was almost the same at both times. Before the first measurement −409. mm/day and before the second measurement −483. mm/day. However, it must be noted that it was raining almost all day on the second sampling day. The long-term mean of precipitation in this area around Cukrák is 587 mm, and the mean annual temperature is 8,6°C (observed between the years 1980 and 2016).

### Reflectance Measurement

Spectral reflectance information was obtained from needles as a bidirectional reflectance factor (BRF). Reflectance was measured between 350 and 2,500 nm by spectroradiometer ASD FieldSpec 4, which was attached to the contact probe (ASD Plant Probe) with the circular field of view of 133 m^2^ (*d* = 11 mm; Schaepman-Strub et al., 2006; Čepl et al., 2018). Spectral reflectance was normalized against a 99% Spectralon white reference panel to produce BRF for each measurement. The scan average on the FieldSpec was set to 15 to avoid foliage overheating, and the integration time was set to 136 ms (Eitel et al., 2006). From each sampled branch (by single tree), several (8–12) shoots with the needles still attached were placed in a Petri dish coated with a spectrally black coating (NEXTEL Velvet-Coating 811-21) with low reflectance, and it was scanned five times. The black surface was set on the samples’ background to eliminate background spectral noise from the surrounding environment. From these measurements, the median for each wavelength was calculated. Reflectance indices were computed using the R package hsdar (Lehnert et al., 2019). The reported indices were pre-selected based on their information value and the predicted heritability and calculated using the R package hsdar (Lehnert et al., 2019).

### Pigment Content Assessment

Needles were cut and inserted along with grinding balls into 2 ml tubes. The samples were freeze-dried by liquid nitrogen and ground for 5 min at 30 Hz in a Mixer Mill MM400 (Retsch technology, Haan, Germany). Freeze–drying and grinding were repeated till the material was powdery. Twenty five milligrams of the powder was weighed into a 2 ml microcentrifuge tube (Eppendorf, Hamburg, Germany), and 1 ml 80% (v/v) acetone and I mg of MgCO3 were added. The samples were vortexed for 30–60 s and then centrifuged (Eppendorf 5424R, Hamburg, Germany) at 13,500 rpm for 10 min. Acetone to 10 ml was added to the supernatant. The absorbance was measured at 646, 663, and 470 nm using a spectrophotometer (Hach Lange DR6000 UV–VIS, Dusseldorf, Germany). The pigment concentration in extracts was calculated according to Lichtenthaler (1987) equations and related to needle dry mass. As mentioned above, the pigment content was assessed only in the August sampling.

### Statistical Analysis

For statistical analysis, native functions of the R software version 3.5.0 (R Core Team, 2022) and the library ASReml for R version 4 (Butler et al., 2017) were used. A univariate linear mixed model was fitted to evaluate all traits of interest with the following terms:


$$y=1\mu +X_{1}\beta_{x}+X_{1}\beta_{y}+Zc+e$$

where *y* corresponds to the data vector; *μ* is the overall mean effect; *β*_*x*_ and *β*_*x*_ are fixed effects associated with orthogonal polynomials of second-order; *c* is the clonal effect, with *c* ~ MVN(**0**, σ^2^_c_*I*_*c*_); *e* is the random vector of errors, with *e* ~ MVN(**0**, *R*). The letters *1*, *X*, and *Z* designate a vector of ones and incidence matrices for associated fixed effects and random effects, and *I*_*c*_ is an identity matrix of order c. *R* is a matrix of variance–covariance of errors for each field position. Two forms of *R* matrix were evaluated, one considering independent errors, i.e., *R* = σ^2^*I*_*n*_ and another based on a separable first-order autoregressive process (AR1) in rows and columns, for which the *R* matrix is *R* = $\sigma^{2}$ [AR1(*p*_col_) ⊗ AR1(*p*_row_)]; where $\sigma^{2}$ are the residual variance, and AR1(*p*_col_) and AR1(*p*_row_) represent a first-order autoregressive correlation matrix. Several models were fitted, considering independent or autoregressive errors and with or without polynomial functions. The best model was selected based on likelihood ratio tests (LRT) and approximated *F*-tests (Isik et al., 2017).

Broad-sense heritability (*H*^2^) was estimated for each response variable based on the following formula: *H*^2^ = *σ*^2^_c_/(*σ*^2^_c_ + *σ*^2^), where *σ*^2^*c* is the genetic variation attributed to represented clones; approximated standard errors were obtained using the Delta method.

We ran an extended bivariate model to estimate the additive correlation between particular traits based on stacking both traits. The model formulae used was:


$$y=X_{1}t+X_{1}t\beta_{x}+X_{2}t\beta_{y}+Z_{1}tc+Z_{2}u+e$$

where *y* corresponds to the data vector of stacked response; *t* is the fixed effect of trait; *tβ*_*x*_ and *tβ*_*x*_ are fixed effects of polynomials of second-order nested within trait; *tc* is the clonal effect nested within trait, with *tc* ~ MVN(**0**, *G*_*c*_ ⊗ *I*_*c*_); *u* is the random vector of experimental units (trees), with *u* ~ MVN(**0**, σ^2^*uI*_*u*_); *e* is the random vector of errors, with *e* ~ MVN(**0**, *R*), where *R* is a block diagonal matrix with a separable first-order autoregressive process as described earlier for each of the traits. Also, *G*_*c*_ is a 2 × 2 variance–covariance matrix with clonal variances on the diagonal and a genetic covariance between traits on the off-diagonal (i.e., unstructured). The random effect *u* allows for specifying correlation between observations (i.e., across traits) that belong to the same tree.

## Results

### Reflectance in the Two Different Sampling Periods

**Figure 1:**
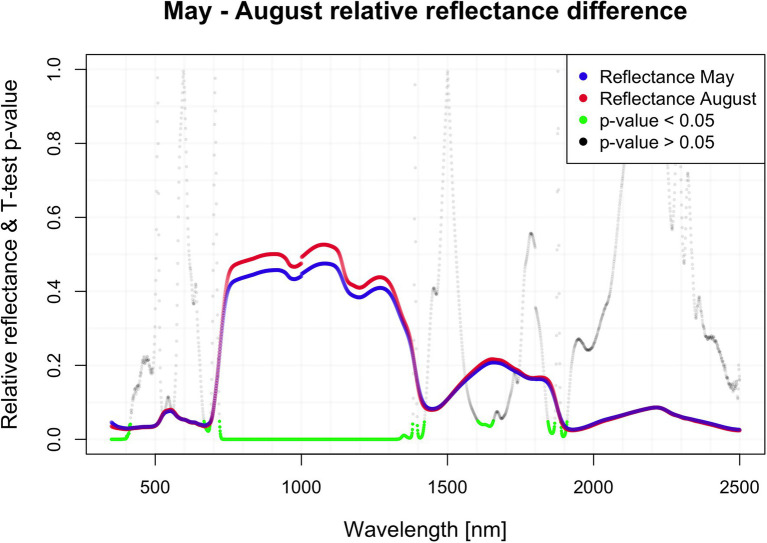
Seasonal difference in hyperspectral reflectance.

There were significant differences (value of *p* < 0.05) in mean reflectance between the two samplings in May and August despite its restriction to several distinct regions, with major differences between 700 and 1,300 nm (NIR plateau). The biggest difference (nearly 5%) was found around 1,050 nm (Figure 1). There were wide regions of wavelengths, at which the reflectance in both respective months significantly differed based on the pairwise t-test. Regions with value of *p* < 0.05: 350–415, 667–668, 720–1,421, 1,604–1,657, 1,844–1,867, and 1,888–1,909. The biggest difference in reflectance was recorded between 720 and 1,421 nm, encompassing the NIR part of the spectra.

### Differences in Reflectance, Pigments, and Growth Traits Between Ecotypes

**Figure 2:**
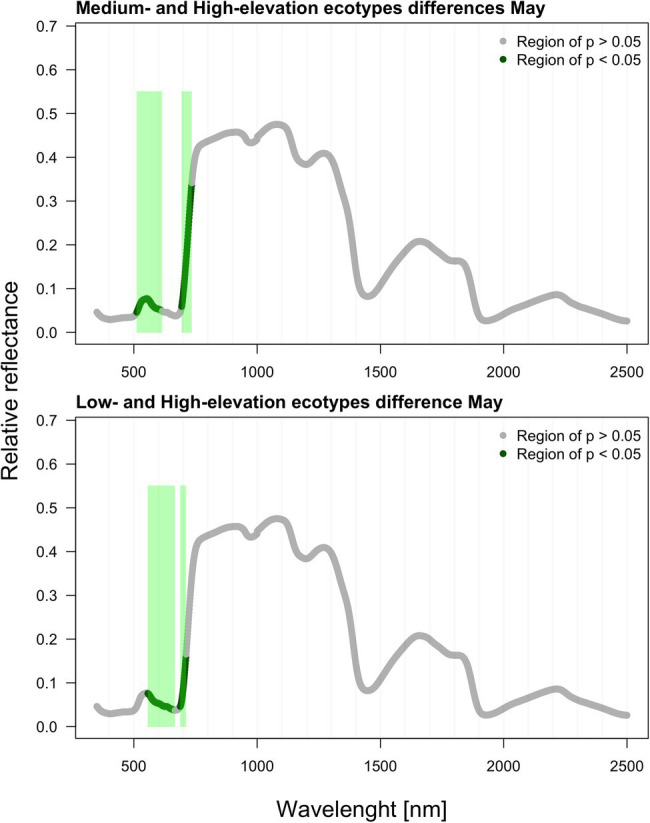
Differences among ecotypes in hyperspectral reflectance recorded in May.

**Figure 3:**
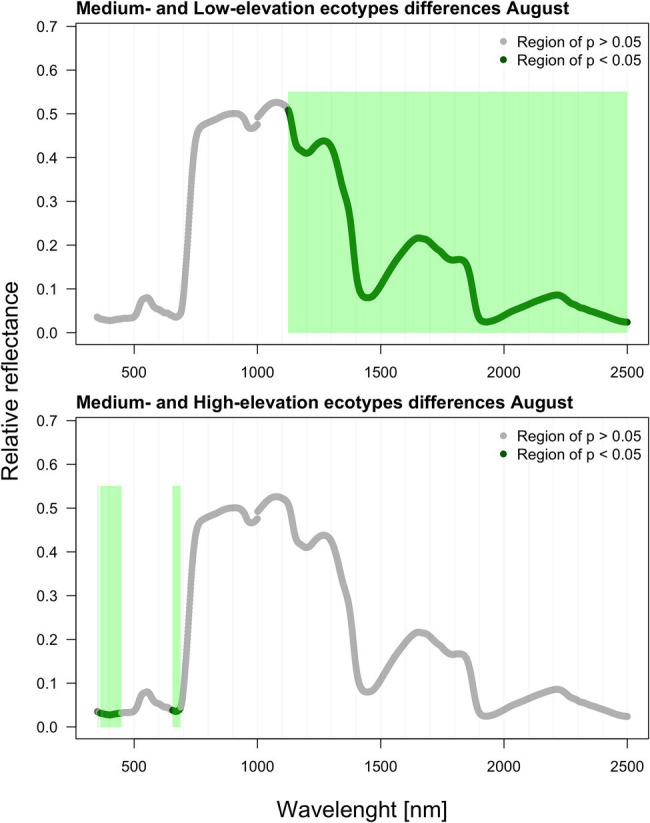
Differences among ecotypes in hyperspectral reflectance recorded in August.

**Figure 4:**
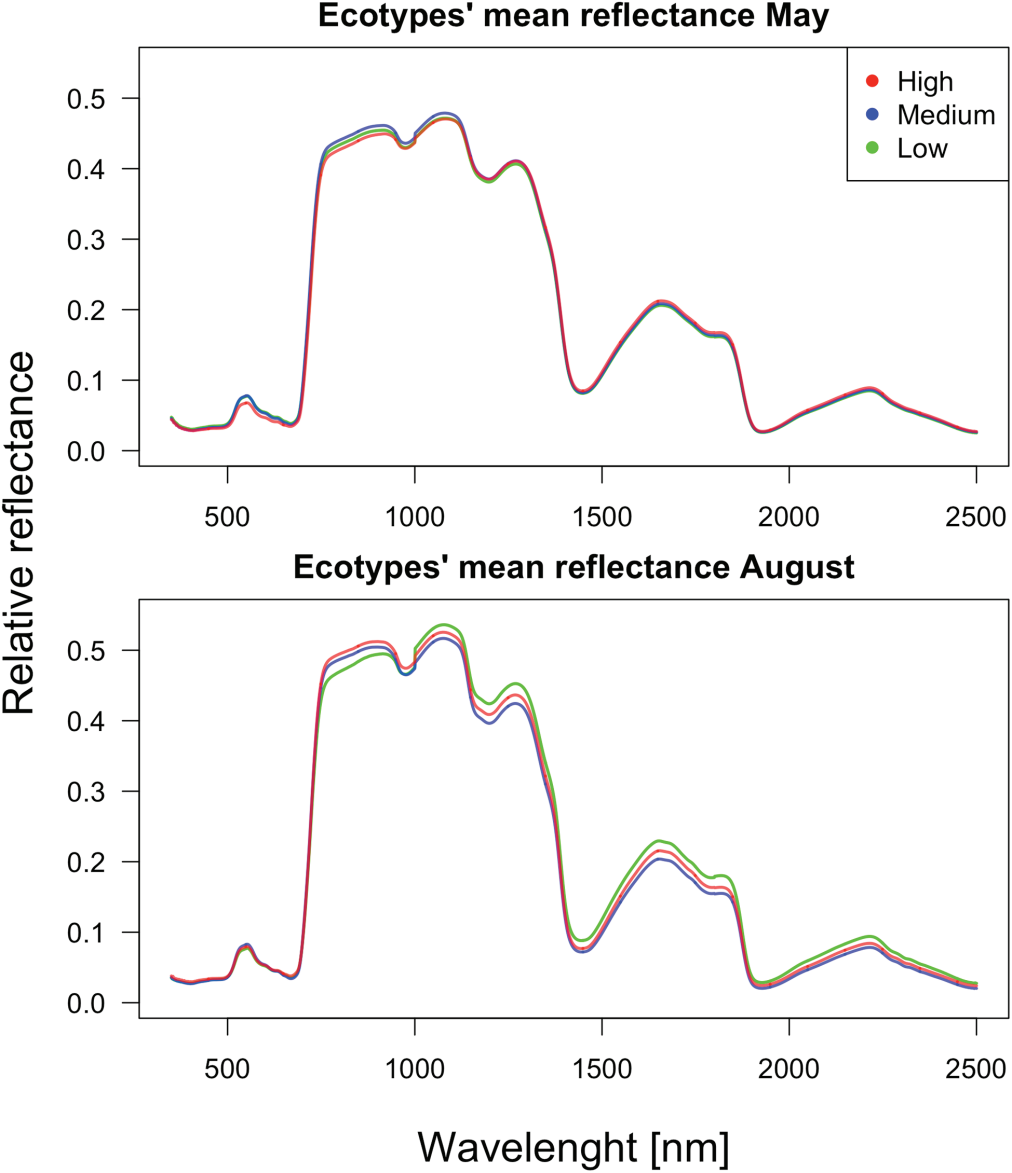
Mean reflectance for each ecotype in May and August.

As verified by multiple comparisons (LSD: least square differences), there were significant (value of *p* < 0.05) differences among ecotypes in reflectance, although this was mostly restricted to specific parts of the spectrum. Differences were generally more pronounced within the second sampling period in August. However, the pairwise differences between ecotypes are month-specific (Figures 2, 3). To further illustrate the variation among ecotypes, we calculated the mean reflectance for each in May and August (Figure 4). The common differences between medium-elevation and high-elevation ecotypes occurred in the reflectance measured in both months. These pairwise differences were situated mostly within the VIS and RE spectral regions: green region (aka green bump) and red-edge in May (Figure 2); and blue and red region in August (Figure 3). Comparing spectral regions distinguishing medium-elevation and high-elevation ecotypes, the ranges of significant difference shifted towards lower wavelengths in August, In May, we found a significant difference in reflectance between the low-elevation and high-elevation ecotypes of Norway spruce, particularly between wavelength 600 and 700, including the red edge (Figure 2). In August, the differences between medium-elevation and high-elevation ecotypes were still present, however, in shorter wavelengths than in May (Figure 3). The low-elevation and high-elevation ecotypes did not show significant differences in reflectance in August. During the August sampling, we recorded a strongly significant difference between medium-elevation and low-elevation ecotypes in reflectance across the NIR and shortwave infrared (SWIR) spectrum (1,150–2,500 nm).

The descriptive statistics for all studied parameters (including photosynthetic pigments and growth traits) are summarized in Table 1. The pairwise differences between ecotypes concerning all studied parameters are summarized in Table 2. We found a significant difference in ecotypes (medium vs. low-elevation) in all three pigments. It should be emphasized that the photosynthetic pigments were extracted only within the August sampling. Thus, the pairwise differences in pigment content are relevant only concerning the reflectance acquired in August. The ecotypes were also significantly different in total height and DBH. In this case, these significant differences were detected among both combinations with the high-elevation origin. The significant differences in pairwise combinations, manifested by the lowest value of *p*s, were in the crown length. In HC, medium elevation vs. high elevation was exactly on the verge of a significant difference (value of *p* = 0.055).

**Table 1:** Descriptive statistics in growth traits and pigment content in various Norway spruce ecotypes.

Ecotype	DBH (m)		H (m)		HC (m)		LC (m)		CHL_a (mg.g^−1^ dry mass)		CHL_b (mg.g^−1^ dry mass)		CAR (mg.g^−1^ dry mass)	
	Mean	SD	Mean	SD	Mean	SD	Mean	SD	Mean	SD	Mean	SD	Mean	SD
Low	32.23	8.09	20.06	3.65	8.68	1.37	12.19	1.75	3.35	0.97	1.22	0.41	0.61	0.18
Medium	33.58	7.27	20.66	2.86	8.52	1.59	12.28	2.44	2.85	0.81	1.02	0.31	0.53	0.13
High	26.51	9.52	16.76	5.11	8.48	3.92	6.59	2.97	3.28	0.52	1.17	0.14	0.59	0.08

**Table 2:** Differences among ecotypes in pigment content and growth.

Differences in ecotypes				
	Ecotypes	Medium vs. low	Medium vs. high	Low vs. high
CAR	Diff.	−0.083	−0.058	0.025
	Std. diff.	0.042	0.056	0.058
	*t*-value	−1.988	−1.033	0.424
	*p*	0.028^*^	0.155	0.337
Chl-a	Diff.	−0.514	−0.353	0.162
	Std. diff.	0.257	0.347	0.360
	*t*-value	−1.999	−1.016	0.449
	*p*	**0.0273** ^*^	**0.159**	**0.328**
Chl-b	Diff.	−0.169	−0.147	0.022
	Std. diff.	0.099	0.132	0.138
	*t*-value	−1.716	−1.108	0.165
	*p*	**0.048** ^*^	**0.138**	**0.435**
H	Diff.	−0.225	3.664	3.890
	Std. diff.	0.760	1.053	1.268
	*t*-value	−0.297	3.4800	3.069
	*p*	**0.384**	**0.000** ^***^	**0.001** ^**^
DBH	Diff.	1.289	6.486	5.197
	Std. diff.	1.725	2.312	2.812
	*t*-value	0.747	2.805	1.848
	*p*	**0.228**	**0.003** ^**^	**0.033** ^*^
HC	Diff.	−0.192	−0.950	−0.758
	Std. diff.	0.391	0.592	0.694
	*t*-value	−0.490	−1.604	−1.092
	*p*	**0.312**	**0.055**	**0.138**
LC	Diff.	0.049	5.155	5.106
	Std. diff.	0.588	0.904	1.055
	*t*-value	0.083	5.705	4.838
	*p*	**0.467**	**0.000** ^***^	**0.000** ^***^

*Reported values based on multiple range tests*.

*Number of asterisks corresponds to significance levels*.

### Phenotypic Correlations Between Hyperspectral Reflectance, Pigment Content, and Growth

**Figure 5:**
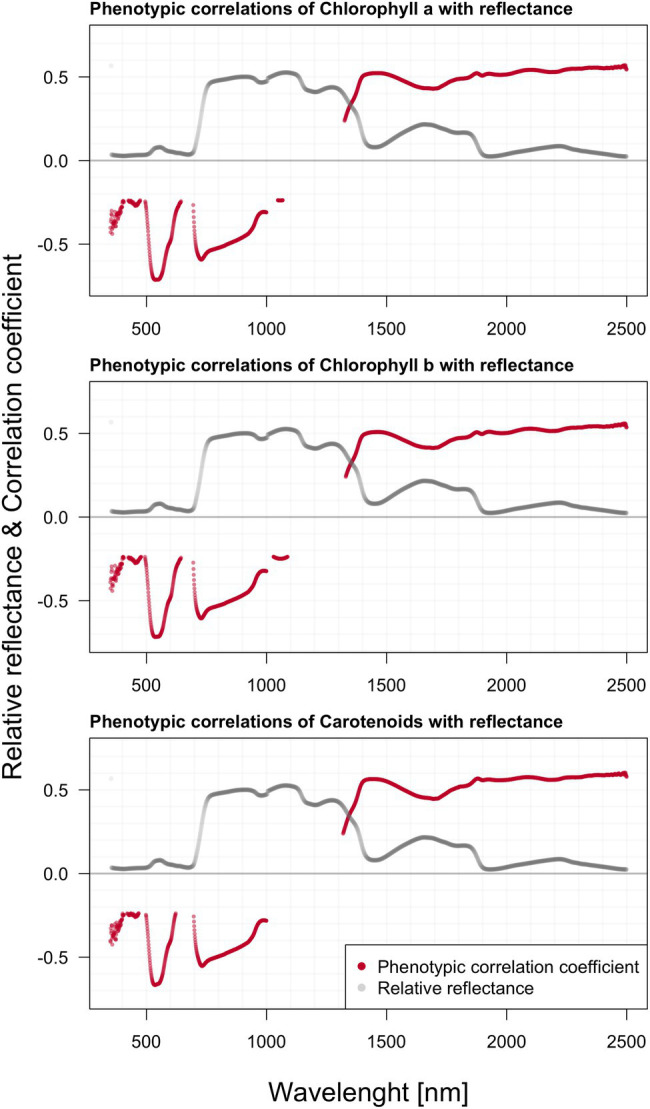
Phenotypic correlations among hyperspectral reflectance and individual photosynthetic pigments content; reported values based on Pearson’s correlation coefficients.

**Figure 6:**
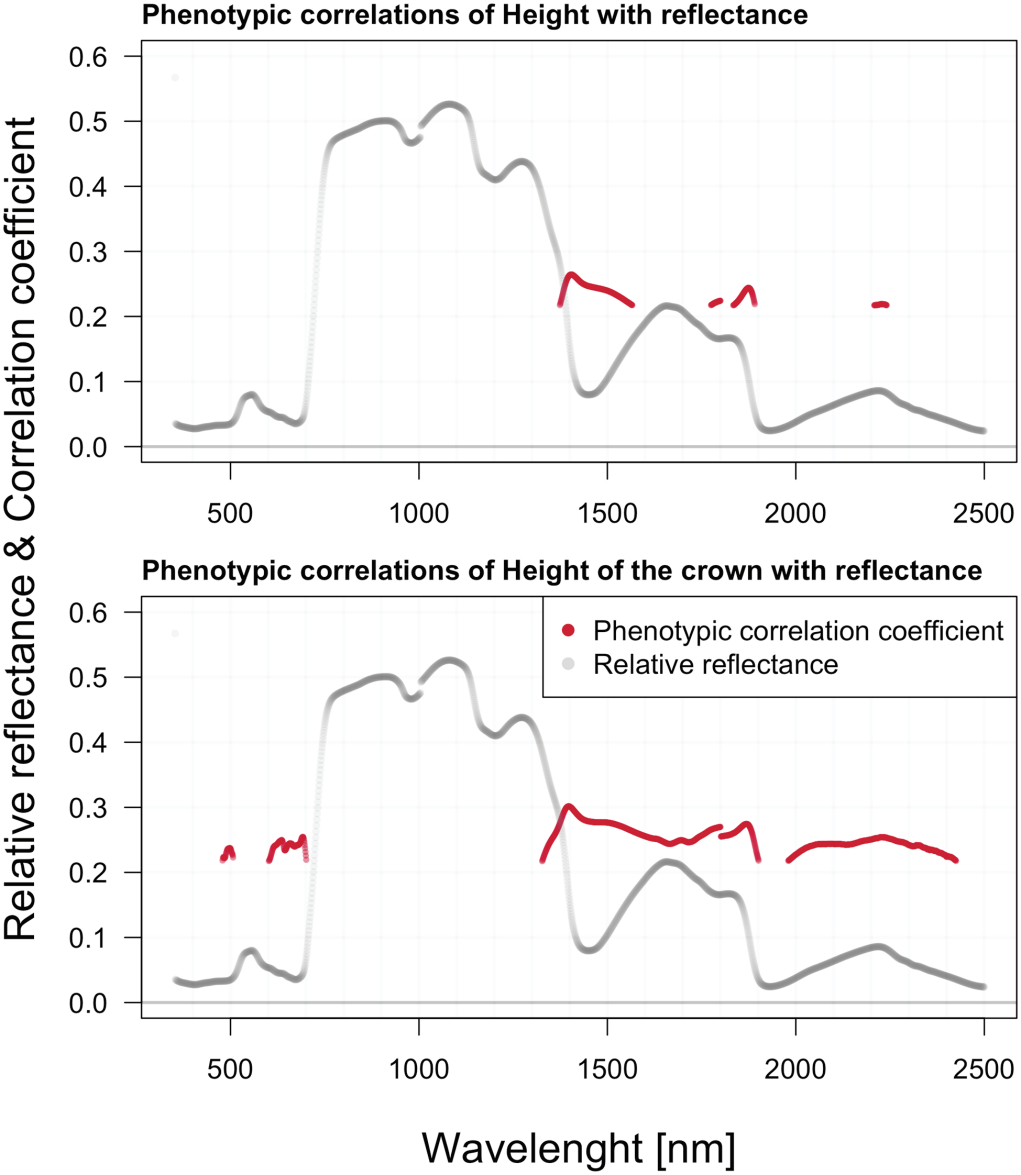
Phenotypic correlations among hyperspectral reflectance and selected growth parameters; reported values based on Pearson’s correlation coefficients.

We found significant phenotypic correlations between spectral reflectance and all three extracted photosynthetic pigments (Figure 5). All three photosynthetic pigments exhibited relatively strong correlations with spectral reflectance, especially around 500, 700, and above 1,300 nm. Generally, the correlation appeared to be strongly negative within the green part of the spectrum, shifting to strong positive correlation values beyond 1,300 nm. The two charts within Figure 6 depict the correlation between reflectance, the trees’ total height, and the crown height. Correlation with HC is more visible as the significant regions stretch across a wider spectrum, especially above 1,300 nm. Further, there was also a prominent position of the green range and far-red region in the vicinity of the red edge.

### Phenotypic Correlations Between the Heritable Hyperspectral Indices, Photosynthetic Pigments, and Measured Growth Traits

In the present study, we used commonly used vegetation indices with a different purpose than usual. Our intention was not to use VIs as predictors of needle biophysical traits, but simply as heritable traits derived from hyperspectral reflectance. Therefore, we selected indices showing the highest estimated heritability (tested by LRT). Later, we studied the phenotypic correlations among those heritable indices, growth parameters, and photosynthetic pigments (Table 3). All reflectance indices (except TCARI2 against carotenoid content) exhibited significant (positive or negative) phenotypic correlations with all three pigments content. However, the *R*^2^ for linear relationships between heritable indices and photosynthetic pigments are rather low (the highest *R*^2^ = 0.59 and 0.511 were found for chlorophylls and DWSI4 and GI, respectively). Overall height, the height of the crown, and the LC exhibited either much lower or zero correlations with the reflectance indices.

**Table 3:** Phenotypic correlations among the hyperspectral indices, photosynthetic pigments, and measured growth traits.

	CRI2 (Gitelson et al., 2003)		DWSI4 (Apan et al., 2004)		GI (Smith et al., 1995)		SR3 (Gitelson and Merzlyak, 1997)		SR4 (McMurtrey et al., 1994)		SR5 (Chappelle et al., 1992)		TCARI2 (Wu et al., 2008)	
	*r*	SE	*r*	SE	*r*	SE	*r*	SE	*r*	SE	*r*	SE	*r*	SE
DBH	−0.002	0.111	0.072	0.111	0.073	0.111	−0.073	0.111	0.137	0.110	−0.158	0.110	0.116	0.110
Height	0.161	0.110	−0.119	0.111	−0.115	0.111	−0.106	0.111	0.007	0.112	−0.016	0.112	**0.220**	0.109
HC	−0.031	0.112	−0.085	0.111	−0.085	0.111	**−0.258**	0.108	−0.019	0.112	0.023	0.112	**0.332**	0.105
LC	**0.221**	0.109	−0.095	0.111	−0.090	0.111	0.032	0.112	0.021	0.112	−0.034	0.112	0.063	0.112
Chl-a	**0.586**	0.099	**−0.722**	0.085	**−0.715**	0.085	**0.453**	0.109	**−0.626**	0.095	**0.678**	0.090	**−0.296**	0.117
Chl-b	**0.590**	0.099	**−0.722**	0.084	**−0.717**	0.085	**0.460**	0.109	**−0.636**	0.094	**0.688**	0.089	**−0.294**	0.117
Car	**0.582**	0.099	**−0.705**	0.087	**−0.698**	0.088	**0.399**	0.112	**−0.583**	0.099	**0.628**	0.095	−0.236	0.119

*Reported values based on Pearson correlation coefficients—SEs in brackets*.

### Genetic Correlations Among Growth Traits: Pigments and Reflectance

Further, we report the genetic correlations based on complex mixed linear models. This approach enabled us to reveal the intricate patterns of genetic correlation (based on clonal attributes) among various traits. We did not find any significant genetic correlations among spectra and growth on the material sampled in May (also, photosynthetic pigments were not extracted in May sampling). Contrastingly, significant genetic correlations were found among many observed parameters in the August sampling date (Table 4). As expected, genetic correlations are generally high when co-dependent growth traits are being observed. This was also confirmed in our experiment. Similarly, photosynthetic pigments show maximum genetic correlations among each other. A similar pattern could be seen concerning phenotypic correlations (results not shown). In Table 3, the genetic correlation between all available measured parameters can be observed. The strongest positive correlations exhibit the photosynthetic pigments across their pairwise comparison. Growth parameters show strong positive correlations, especially between DBH, total height, and crown length. Interestingly, all three pigments were relatively (but not significantly according to rigorous LRT) correlated to the crown’s length (0.58–0.78), with the highest value between carotenoids and LC.

**Table 4:** Estimated genetic correlations among photosynthetic pigments content and growth traits.

Trait	Trait	Genetic correlation	SE
Height	DBH	0.999	B
Height	Crown height	**0.816**	0.065
Height	Crown length	0.998	B
DBH	Crown height	**0.367**	0.114
DBH	Crown length	0.998	B
Chl-a	Car	0.998	B
Chl-a	Chl-b	0.999	B
Car	Chl-b	0.999	B
Chl-a	Crown length	**0.702**	0.279
Chl-b	Crown length	0.590	0.319
Car	Crown length	**0.787**	0.237

B denotes the boundary effect of a model. Bold values passed the LRT test.

**Figure 7:**
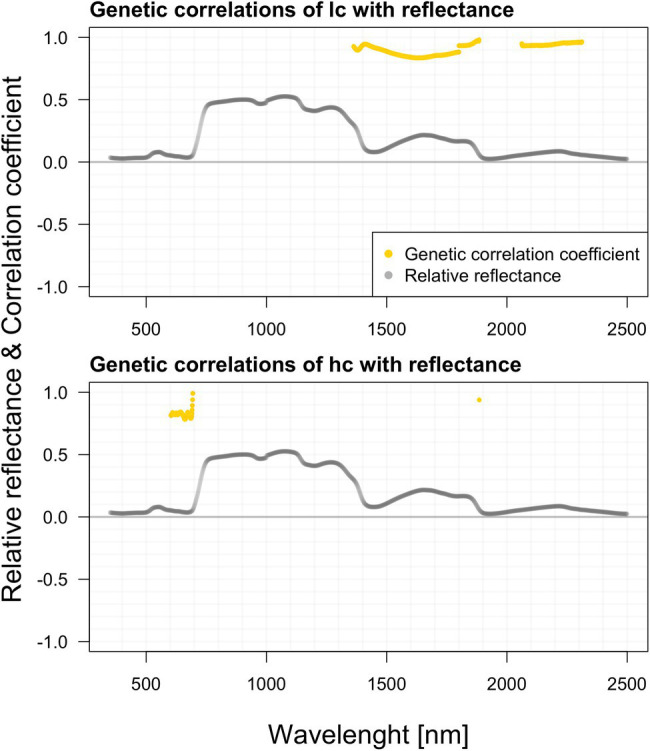
Genetic correlations among hyperspectral reflectance and selected growth parameters.

We did not estimate any significant genetic correlations between hyperspectral data, height, and DBH. Genetic correlation of the length of a crown (LC) with reflectance is significant in a relatively wide range of the spectrum, especially within the region 1,360–1,900 nm (minimum value of 0.89 with SE 0.23) and in the region 2,050–2,420 nm (min 0.931 with SE 0.2287) a strong positive correlation was recorded. Genetic correlation in the crown (HC) height with reflectance is comparatively less abundant across the spectra than the crown’s length (LC). Interesting is the visible correlation peak occurring between 600 and 700 nm in the vicinity of the red edge. These correlations are highlighted in Figure 7.

**Figure 8:**
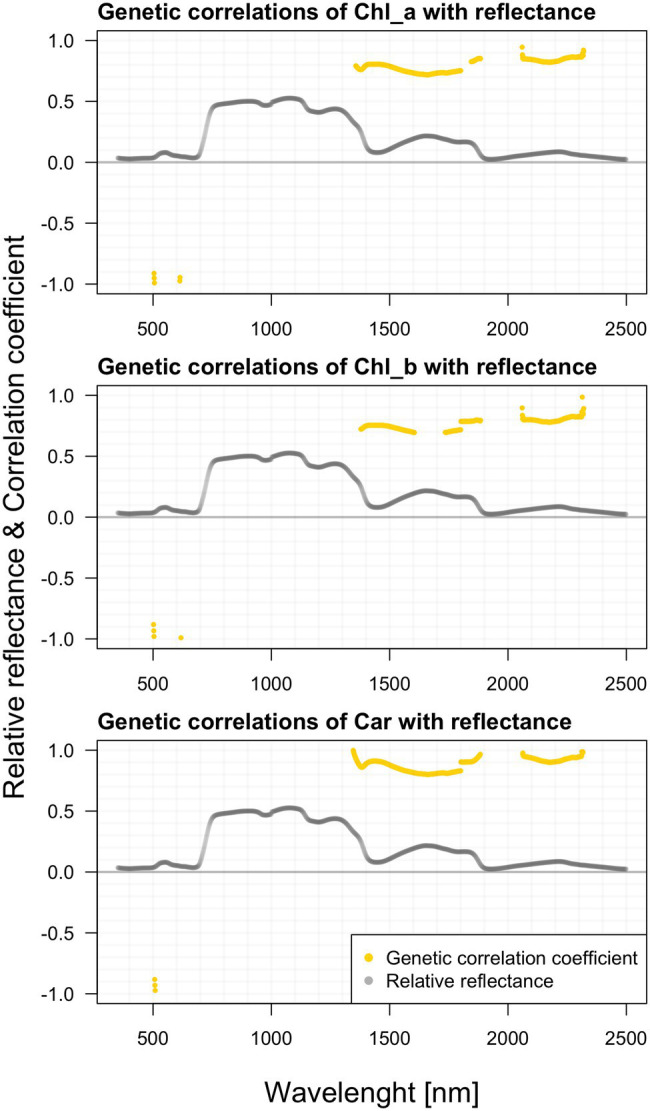
Genetic correlations among hyperspectral reflectance and individual photosynthetic pigments content.

In contrast to growth traits, we found significant genetic correlations between the raw spectral data and all photosynthetic pigments (Figure 8). Like the phenotypic relations depicted earlier, the visible differences among individual pigments are rather marginal. Correlations of chlorophyll a with reflectance are significant mainly within the NIR and SWIR. There are substantial correlations in 1,360–1,800 and 2,050–2,240 nm. Along the green-light region from 500 to 600 nm, the contrasting strong negative correlations below −0.900 occur, following the similar pattern observed in the above-illustrated phenotypic correlations. Correlations between chlorophyll b and reflectance are negative again in the visible parts of the spectrum and positive in the far regions. Carotenoids do correlate with reflectance in an almost identical pattern as chlorophyll b. We found strong negative correlations (below −0.880) in the region 507–518. In 1315–1885 and 2,060–2,320 nm, we found other significant ranges.

### Genetic Correlations Among Growth Traits: Pigments and Reflectance Indices

Traditionally, the spectral information has rendered indices pre-selected due to their specific properties connected to stress relation or health status determining capacity. With this in mind, we chose those indices from the original candidate set contained within the R package hsdar (Lehnert et al., 2019) that were heritable for the subsequent bivariate analysis, which allowed us to estimate genetic correlations with other traits of interest. This selected subset is listed in Table 5. The highest heritability values were recorded in SR3. In this case, almost 50% of these indices’ variation is controlled by genetics (attributed to clonal variation) in our experimental plot. The relatively high standard error values can be attributed to the small sample size used in this study.

To picture the complex relationships among various traits, further elaborated within genetic correlations, we also present the estimated heritability in all measured traits (Table 5). Finally, genetic correlations between multiple reflectance indices and other recorded parameters were calculated in the bivariate models (Table 6). There are relatively high values of genetic correlations with photosynthetic pigments. The highest genetic correlation (0.99) was found between chlorophyll b, chlorophyll a, and the CRI2 index. CRI2 is also highly correlated with chlorophyll b (0.97).

**Table 5:** Estimated heritability of reflectance indices, photosynthetic pigments, and growth traits.

Refl. Index/Pigment/Growth	Heritability	SE
CRI2	0.30	0.22
DWSI4	0.31	0.15
GI	0.32	0.15
GMI1	0.46	0.19
SR3	0.46	0.19
SR4	0.39	0.15
SR5	0.39	0.15
TCARI2	0.39	0.19
Chlorophyll a	0.31	0.14
Chlorophyll b	0.36	0.13
Carotenoids	0.29	0.11
LC	0.36	0.12
HC	0.41	0.07
Height	0.41	0.14
DBH	0.33	0.06

**Table 6:** Genetic correlations among the hyperspectral indices, photosynthetic pigments, and measured growth traits.

VI calcul.	R_760_/R_700_ - 1	R_550_/R_680_	R_554_/R_677_	R_750_/R_550_	R_700_/R_670_	R_675_/R_700_	3*[(R_750_-R_705_)-0.2*(R_750_-R_550_)*(R_750_/R_705_)]
	CRI2 (Gitelson et al., 2003)	DWSI4 (Apan et al., 2004)	GI (Smith et al., 1995)	SR3 (Gitelson and Merzlyak, 1997)	SR4 (McMurtrey et al., 1994)	SR5 (Chappelle et al., 1992)	TCARI2 (Wu et al., 2008)
DBH	0.189 (0.338)	−0.083 (0.335)	−0.084 (0.332)	0.134 (0.319)	0.145 (0.331)	−0.262 (0.333)	0.182 (0.333)
h	0.292 (0.286)	−0.255 (0.279)	−0.248 (0.277)	−0.068 (0.28)	−0.020 (0.288)	−0.022 (0.294)	0.318 (0.274)
hc	−0.081 (0.310)	−0.172 (0.289)	−0.172 (0.286)	−0.475 (0.242)	−0.021 (0.290)	0.041 (0.295)	**0.659 (0.224)**
lc	**0.720 (0.249)**	**−0.954 (0.184)**	**−0.946 (0.185)**	0.461 (0.304)	−0.720 (0.256)	0.668 (0.276)	−0.155 (0.365)
Chl-a	**0.916 (0.198)**	−0.999 (0.000)*^b^*	−0.998 (0.000)*^b^*	**0.747 (0.233)**	−0.999 (0.000)*^b^*	0.999 (0.000)*^b^*	**−0.631 (0.276)**
Chl-b	**0.967 (0.201)**	−0.999 (0.000)*^b^*	−0.999 (0.000)*^b^*	**0.844 (0.232)**	−0.999 (0.000)*^b^*	0.999 (0.000)*^b^*	**−0.746 (0.276)**
Car	0.999 (0.000)*^b^*	−0.999 (0.000)*^b^*	−0.999 (0.000)*^b^*	NA	−0.999 (0.000)*^b^*	0.999 (0.000)*^b^*	−0.519 (0.284)

*The significant, further reported values are in bold; the SE in brackets; the upper index *b* denotes the boundary effect connected to the model convergence—these values are not fully reliable despite passing the LRT test*.

In contrast to phenotypic correlations among indices and growth, strong negative genetic correlations were found between LC and DWSI4 and GI (−0.95). On the other hand, CRI2 correlated strongly positively with the LC parameter. Lastly, TCARI2 exhibits a strong positive genetic correlation with an HC parameter.

## Discussion

### Seasonal Variation in Hyperspectral Reflectance

It is known that hyperspectral reflectance mirrors the actual conditions in forest stands, and these specific differences are temporal and spatial. We detected a significant difference between the shoot reflectance in May and August in the spectral range from 700 to 1,300 nm.

The mean reflectance curve in both periods has an almost identical shape except for the region between 700 and 1,300 nm. The biggest difference was detected around 1,050 nm. The NIR region responds to many structural parameters in plant tissue (Rock et al., 1994; Slaton et al., 2001; Sánchez et al., 2012). This detectable difference in NIR may occur since we sampled mostly previous year’s needles in May, whereas we measured the current needles during the August sampling. In other words, the higher NIR reflectance from August samples may reflect the different structural parameters of current-years vs. previous-season needles. A similar trend in reflectance decrease with needle aging was reported for Norway spruce (Hovi et al., 2017; Lhotáková et al., 2021). The apparent inconsistency in the age of needle samples was since some trees did not produce new shoots in the 2020 season in the reach of our sampling device (up to 8 meters above ground). However, the youngest available needles were supposed to be the most physiologically active as reported for various conifers (Bernier et al., 2001; Jensen et al., 2015) and thus suitable for revealing differences in needle physiological (pigments) and optical traits among the studied ecotypes.

Needle water content also affects NIR reflectance: directly in water absorption bands centered around 970, 1,200, 1,450, and 1930 nm (some spectral intervals of significant differences among sampling dates correspond to these spectral regions); and indirectly due to decreasing cell turgor during dehydration (Peñuelas and Inoue, 1999), which could be included in the structural effect mentioned above. Although we did not quantify water content in needles, we suppose it is directly influenced by available soil moisture. Despite the higher average precipitation recorded in August (4.3 mm vs. 2.6 mm in May), available moisture was perhaps much lower in late summer. The mean temperature was noticeably higher in August (20.9°C) than in May (12.5°C). Thus, the higher temperatures led to increased evapotranspiration (the mean values may be biased by the thunderstorm that preceded our August sampling). These specific circumstances may have indirectly affected needles’ spectral properties and contributed to differences in NIR reflectance between May and August.

### Differences Among Ecotypes

The typical morphological features of respective ecotypes were retained in the studied common garden. We assumed the adaptation to the original climatic conditions was genetically determined. These assumptions were supported by preliminary SNPs data cluster analysis (Čepl et al., 2020). Although there is no information about distinguishing Norway spruce ecotypes from spectral reflectance neither at canopy nor at needle level, we expected these adaptations to be reflected in variable hyperspectral reflectance of young shoots. We based our hypothesis on previous findings in *Quercus* populations, which were more distinguishable by hyperspectral reflectance than individual functional leaf traits (Cavender-Bares et al., 2016).

Recently, multivariate statistical approaches that work with continuous spectra are frequently used in laboratory and image spectroscopy (Deepak et al., 2019; Verrelst et al., 2019; Lhotáková et al., 2021). We also tested principal component analysis and random forest classification for distinguishing Norway spruce ecotypes in the present study. However, the results were not convincing (data not shown). High variability in reflectance spectra and broad spectral regions with high spectral similarity could fail to differentiate the ecotypes. Therefore, we applied a pairwise comparison of individual wavelengths.

We recorded the significant differences among Norway spruce ecotypes in reflectance. However, they were mostly limited to specific and quite narrow regions of the spectra and depended on the sampling month. These reflectance differences among represented ecotypes were more frequent and pronounced in the second sampling period (in August). In May, the differences among two ecotype pairs (medium-elevation and high-elevation; low-elevation and high-elevation) were concentrated in the VIS part of the spectrum (green and red region) and partly red edge, which makes sense considering the accumulation of needle chlorophyll with aging. This process may not be synchronized among the ecotypes, even though they grow in the same environment. We can only hypothesize about this cause as we did not sample needles for biochemical analyses in May. In the pairwise comparison between medium-elevation and high-elevation ecotypes, the ranges of significant difference shifted back towards the visible parts of spectra, corresponding to the putative red edge shift after a prolonged drought (Curran et al., 1990) caused by heatwaves. At the level of cell membrane stability, the high-elevation Norway spruce ecotype from Germany was less thermotolerant than its low-elevation counterpart (Valcu et al., 2008); however, the relation of thermotolerance to reflectance spectra was not investigated.

Conversely, in August, the biggest differences were found between low-elevation and medium-elevation ecotypes covering the broad part of the NIR plateau and SWIR. We attribute the differences in these spectral regions rather to low water content in medium-elevation ecotype (having the lower reflectance). The peak of heritable variation in the red edge described by Čepl et al. (2018) is here further documented on a much broader scale of within-species genetic variation inherent to various origins.

Further, we explored the potential differences among represented Norway spruce populations concerning photosynthetic pigments content and various growth traits. All photosynthetic pigments behaved equally, showing significantly lower contents in the medium-elevation ecotype than the low-land ecotype. Later the ecotypic variation within pigments emerged only in medium- vs. low-elevation comparison (in contradiction to spectral data), which can be attributed to the under-representation of the high-elevation source. We could speculate that the trait is more sensitive to sample size within these multiple comparisons.

The ecotypes were also significantly different in total height and DBH. These significant differences were detected among both combinations with high-elevation origin. Morphologically, the high-elevation origin is unique, mirrored in any pairwise comparison highlighting the difference. The two significant differences across all pairwise combinations connected with very low value of *p*s were found in the LC parameter. This major difference could be attributed to visible differences in crown morphology among respective ecotypes. These habitual characteristics were retained in the clonal trial and further contributed to other physiological differences. In HC, medium elevation vs. high elevation is exactly on the verge of a significant difference (value of *p* = 0.055). We concluded that this growth trait is less informative about the recent increment and health status and does not contribute much to the observed ecotypic variation.

Our study showed several potentially adaptive traits that help characterize Norway spruce populations from cold mountain environments. It has been documented that populations originating from colder high-elevation sites often differed per unit change in altitude or mean annual temperature more than low elevation populations did (Oleksyn et al., 1998).

We analyzed the ecotypic variation of Norway spruce elevation forms grown in natural conditions of a common-garden experiment (i.e., clone bank). This makes our findings unique, as methodically similar research is predominantly performed under controlled conditions in greenhouse or growth chambers (e.g., Eldhuset et al., 2013; Sena et al., 2018) while studies of Norway spruce grown under natural conditions are scarce (e.g., Yakovlev et al., 2008). Tomášková et al. (2021) hypothesized that the high elevation spruce ecotype would strive less compared to the medium and lowland one after a prolonged period of drought in 2018. The experiment confirmed high adaption potential for high-mountain spruce ecotype planted in low elevation based on variable chlorophyll a fluorescence (Tomášková et al., 2021). Our results support the adaptation potential of high-elevation ecotype at the level of photosynthetic apparatus, as the contents of photosynthetic pigments were not significantly decreased compared to medium- and low-elevation ones. Similarly, the results of spectral analyses support the adaptation potential of high-elevation ecotype, as the differences in hyperspectral reflectance were restricted to specific parts of the spectrum, and the multivariate analyses failed to distinguish the ecotypes.

### Correlations Between Hyperspectral Reflectance, Pigment Content, and Various Growth Parameters

We presented phenotypic correlations between photosynthetic pigments and reflectance in single wavelengths in the measured spectral range. Not surprisingly, chlorophylls and carotenoid content correlate mostly with VIS wavelengths, which correlate mostly with VIS wavelengths, determined by their molecular structure and specific absorption features (Ustin et al., 2009). Similarly, Schlerf et al. (2010) evaluated the information content of spectral reflectance (laboratory and airborne data) for the estimation of needle chlorophyll concentration in Norway spruce (*Picea abies* L. Karst.) needles. The wavebands selected in the regression models to estimate chlorophyll concentration were typically located in the red edge region and near the green reflectance peak. Even though pigments do not absorb NIR wavelengths, NIR reflectance is not uncommon to contribute to chlorophyll retrieval models (Lhotáková et al., 2021).

All photosynthetic pigments show a substantial positive genetic correlation between 1,357–1,800 and 1,845–2,240 nm. Along the green-light region from 504 to 614 nm, the contrasting strong negative correlations below −0.91 occur, following the similar pattern observed in the above-illustrated phenotypic correlations. We found strong negative correlations (below −0.88) in the regions 507–518 and 585–605 nm. In 1,313–1,884 and 2,060–2,320 nm we found high positive genetic correlations. It seems that this inverted relationship across the spectra might be a general pattern observable in various populations of different species. This phenomenon could be attributed to the presumably positive relationship between water content and pigment content, as both are vital for the striving of the plants. The repeated drops along the reflectance curve are commonly attributed to water content.

In contrast to systematic studies focused on correlating and modeling pigments contents from leaf-level reflectance, the studies relating leaf reflectance to growth traits of forest trees are absent. In crop science, canopy reflectance is commonly used to estimate aboveground biomass or yield (Yang and Chen, 2004; Wang et al., 2017). In forest science, the tree height and aboveground biomass are usually modeled from canopy level lidar data or lidar (Persson et al., 2002), hyperspectral data fusion (Sankey et al., 2017), or UAV-borne image data with a very high spatial resolution (Tao et al., 2021). In contrast, we tested the single wavelength shoot-level reflectance correlation to all three growth traits such as DBH, tree height, crown height, and length. We suppose that the relationships among shoot level reflectance and growth traits would be indirect, reflecting several physiological processes such as photosynthetic capacity, assimilate partitioning into growth, reproduction, and defense (Zhang et al., 2015). However, from presented correlations between the shoot level reflectance and growth traits, the mechanisms behind could not be elucidated. Some of the selected spectral attributes were shown to have significant relationships with growth parameters in phenotypic and genetic correlations. Phenotypic correlation with HC is more visible as the significant regions stretched across a much wider range of the spectrum, especially beyond 1,300 nm. Phenotypic correlation between the HC and blue and red spectral regions could be mediated by chlorophyll absorption in these wavelengths. Further, there is also a prominent position of the green range and far-red region in the vicinity of the red edge. Contrastingly, the height correlates against spectra only within NIR and SWIR.

We did not find any genetic correlations between shoot hyperspectral reflectance and height with DBH. Genetic correlation of the LC with reflectance is significant in a relatively wide range of the spectrum, especially within the region 1,363–1,901 nm (minimum value of 0.89 with SE 0.23) and in the region 1,989–2,438 nm (min 0.93 with SE 0.23) a strong positive correlation was recorded. Genetic correlation in the crown height (HC) with reflectance is comparatively less abundant across the spectra than the LC. There are significant ranges in 1,887–1,897, 1,995–2,057, and 2,322–2,397 nm. But more interesting is the visible correlation peak occurring between 603 and 704 nm in the vicinity of the red edge. We can speculate that these relationships might share a similar background with pigment content genetic correlations. The water content (reflected in NIR and FAR) will likely be positively correlated with the overall photosynthetic capacity of trees, which is further reflected in the LC.

### Reflectance Indices: Their Heritability and Correlations With Growth and Physiological Traits

Spectral reflectance measurement provides a fast, non-destructive method for pigment and other leaf or canopy traits estimation (Sims and Gamon, 2002; Croft et al., 2014). Many spectral indices have been developed (see Footnote 1). All three photosynthetic pigments show significant correlations against the selected reflectance indices in our study. We deliberately chose those established indices, where we estimated the heritable variation. It should be stressed that this broad-sense heritability was based on clonal variation present in our trial. The heritability values presented here are slightly higher than previous experiments with a half-sib or full-sib genetic structure (Čepl et al., 2016, 2018), which allowed the estimates of the narrow-sense heritability. The standard error (SE) values vary across the measured traits strongly connected to the sample size. We can observe that growth traits tend to have lower SE (0.06–0.14) as they comprise the whole common garden. Highly heritable traits can benefit from multivariate analyses as genetic correlations can be estimated to predict the effectiveness of indirect selections on one trait with the primary goal of improving a different trait (Isik et al., 2017). Genetic correlation estimates in population and evolutionary genetics are useful to understand how different traits are genetically related to fitness and how natural selection for higher fitness affects or is constrained by other traits (Isik et al., 2017).

In the preselection of heritable reflectance indices, the majority are simple ratios. Only two out of seven indices are more complex (namely CRI2 and TCARI2). All reflectance indices (except TCARI2 vs. Carotenoids content) exhibit significant positive or negative phenotypic correlations with all three pigments content. A significant negative correlation was recorded in pigments with DWIS4 and GI reflectance indexes (<−0.70). Contrastingly, the highest positive correlation was found between pigments and the SR5 index (0.63). These values follow the correlations reported in previous studies (Datt, 1999; Solovchenko et al., 2010; Hernández-Clemente et al., 2012; Sonobe and Wang, 2017).

Overall, the highest genetic correlation (0.99) was found between Chlorophyll b, chlorophyll a, and the CRI2 index. CRI2 is also highly correlated with Chlorophyll b (0.97). We can conclude that the genetic correlations generally reach higher values but often exhibit higher SE due to their higher sensitivity to sample size.

In Norway spruce, the red edge position can indicate physiological conditions due to air pollution (Campbell et al., 2004). Moorthy et al. (2008) investigated quantitative links among various Chlorophyll concentrations hyperspectral observations of Jack Pine (*Pinus banksiana*) in the forest health context. They concluded that chlorophyll content measurements are useful bioindicators of stand health conditions.

Our evaluation’s novelty lies in the reported phenotypic/genetic correlations of reflectance indices against various growth parameters. This concept is more often seen in crop science, and our results may indicate the potential for forestry and tree improvement. It is more feasible to train and validate prediction models in highly uniform crop fields even across multiple sites. Rather heterogeneous conditions create an obstacle to further applications of these methods in forestry. We see a clear potential for forest trees from the strong and significant genetic correlations we estimated through the specific design utilizing clonal replications. In our study height, the height of the crown and the LC correlate rather poorly with the reflectance indices. CRI2 showed a low positive correlation against LC; TCARI2 exhibits a moderate positive phenotypic correlation with an HC.

In contrast, strong negative genetic correlations were found between LC and DWSI4 and GI (−0.95). Conversely, CRI2 correlated strongly positively with the LC parameter. Lastly, TCARI2 exhibits a strong positive genetic correlation with an HC parameter. It seems that genetic correlations between spectral indices and various growth parameters possibly connected to pleiotropy reflect the actual productivity and health status much more precisely than the simple phenotypic correlations. It is questionable if this is given by the higher precision of spatial modeling, which takes the experimental design and microenvironment into account.

## Data Availability Statement

The original contributions presented in the study are included in the article/Supplementary Material, further inquiries can be directed to the corresponding author.

## Author Contributions

JH and JS coordinated research activities. JS, JH, and JD collected material in the field. ZL, JD, and AK did laboratory work. JS, JH, SG, and JČ built statistical models. JS, JH, JČ, JK, SG, and ZL wrote the manuscript. All authors contributed to the article and approved the submitted version.

## Funding

This work was supported by the National Agency of Agriculture research, Czechia (NAZV) (grant number QK1910480); the project EXTEMIT-K: “Building up an excellent scientific team and its spatiotechnical background focused on mitigation of the impact of climatic changes to forests from the level of a gene to the level of a landscape at the FFWS CULS Prague,” (grant number CZ.02.1.01/0.0/0.0/15_003/0000433) financed by OP RDE; The Ministry of Education, Youth, and Sports program INTER-EXCELLENCE, subprogram INTER-ACTION (grant number LTAUSA19113).

## Conflict of Interest

The authors declare that the research was conducted in the absence of any commercial or financial relationships that could be construed as a potential conflict of interest.

## Publisher’s Note

All claims expressed in this article are solely those of the authors and do not necessarily represent those of their affiliated organizations, or those of the publisher, the editors and the reviewers. Any product that may be evaluated in this article, or claim that may be made by its manufacturer, is not guaranteed or endorsed by the publisher.

## Footnotes

^1^ https://www.indexdatabase.de/
