# Supplementary figures and images for: Revealing the Complex Relationship Among Hyperspectral Reflectance, Photosynthetic Pigments, and Growth in Norway Spruce Ecotypes
Source: Front Plant Sci. 2022 May 30;13:721064. doi: 10.3389/fpls.2022.721064 (PMC9197180; doi:10.3389/fpls.2022.721064)

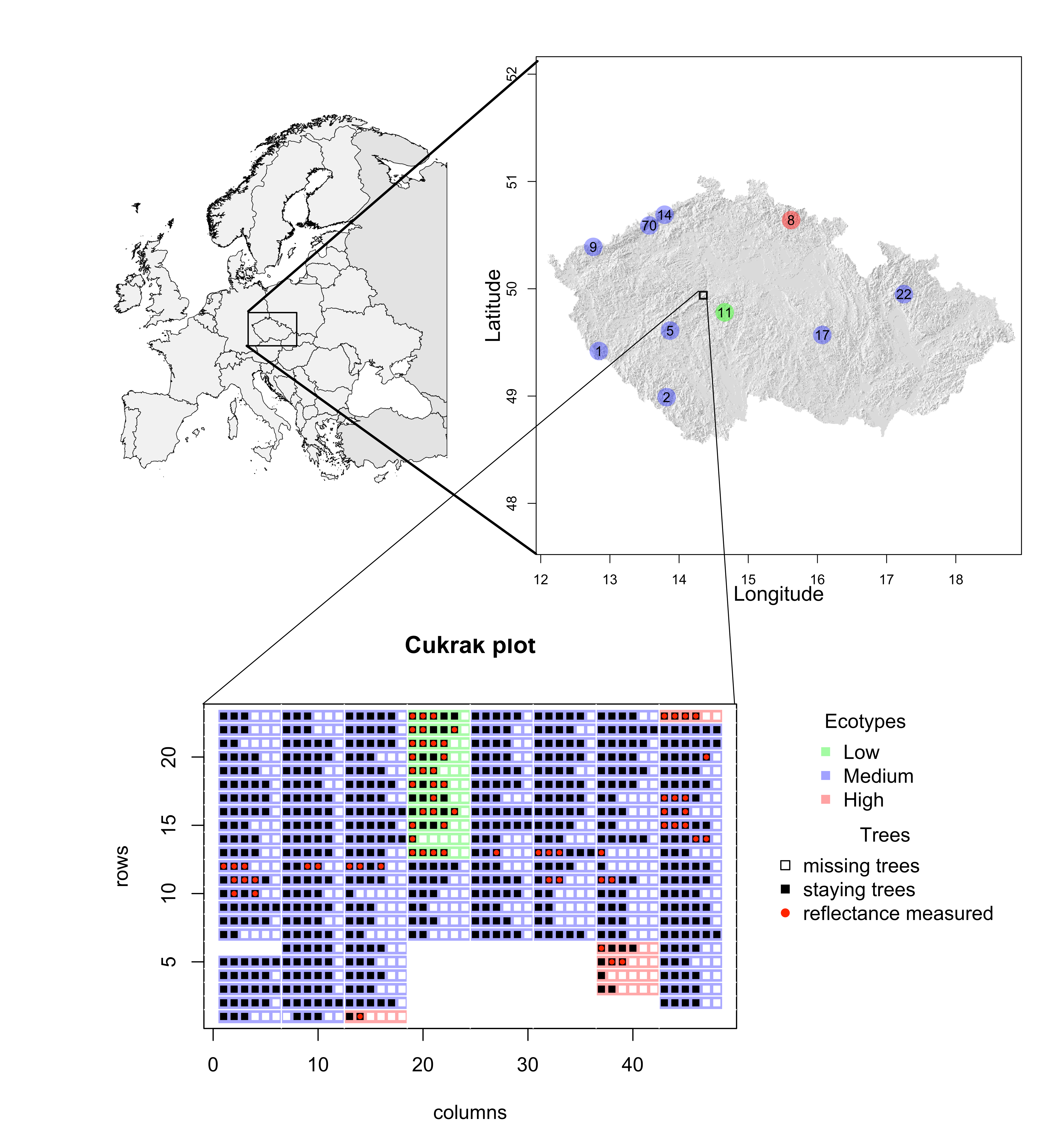

Supplement: Supplementary file 2 [file Image_1.TIF]
